# Identification of bacterial pathogens in sudden unexpected death in infancy and childhood using 16S rRNA gene sequencing

**DOI:** 10.3389/fmicb.2023.1171670

**Published:** 2023-06-15

**Authors:** Lily Gates, Talisa Mistry, Olumide Ogunbiyi, Kerry-Anne Kite, Nigel J. Klein, Neil J. Sebire, Dagmar G. Alber

**Affiliations:** ^1^Infection, Immunity and Inflammation, UCL GOS Institute of Child Health, London, United Kingdom; ^2^NIHR GOSH Biomedical Research Centre, Histopathology Department, Camelia Botnar Laboratories, Great Ormond Street Hospital, London, United Kingdom

**Keywords:** sudden unexpected death in infancy (SUDI), sudden unexplained death in childhood (SUDC), sudden infant death syndrome (SIDS), infection, post-mortem, bacteria, 16S

## Abstract

**Background:**

Sudden unexpected death in infancy (SUDI) is the most common cause of post-neonatal death in the developed world. Following an extensive investigation, the cause of ~40% of deaths remains unknown. It is hypothesized that a proportion of deaths are due to an infection that remains undetected due to limitations in routine techniques. This study aimed to apply 16S rRNA gene sequencing to post-mortem (PM) tissues collected from cases of SUDI, as well as those from the childhood equivalent (collectively known as sudden unexpected death in infancy and childhood or SUDIC), to investigate whether this molecular approach could help identify potential infection-causing bacteria to enhance the diagnosis of infection.

**Methods:**

In this study, 16S rRNA gene sequencing was applied to de-identified frozen post-mortem (PM) tissues from the diagnostic archive of Great Ormond Street Hospital. The cases were grouped depending on the cause of death: (i) explained non-infectious, (ii) infectious, and (iii) unknown.

**Results and conclusions:**

In the cases of known bacterial infection, the likely causative pathogen was identified in 3/5 cases using bacterial culture at PM compared to 5/5 cases using 16S rRNA gene sequencing. Where a bacterial infection was identified at routine investigation, the same organism was identified by 16S rRNA gene sequencing. Using these findings, we defined criteria based on sequencing reads and alpha diversity to identify PM tissues with likely infection. Using these criteria, 4/20 (20%) cases of unexplained SUDIC were identified which may be due to bacterial infection that was previously undetected. This study demonstrates the potential feasibility and effectiveness of 16S rRNA gene sequencing in PM tissue investigation to improve the diagnosis of infection, potentially reducing the number of unexplained deaths and improving the understanding of the mechanisms involved.

## Introduction

Sudden unexpected death in infancy (SUDI) is the sudden and unexpected death of an infant less than 1 year of age. The sudden nature of the death requires extensive investigation including death scene examination, full review of clinical history, and autopsy (Weber et al., 2012; Kennedy, 2016). During the investigation, all possible mechanisms of death are explored, and evidence of a mechanism must be identified for the cause of death (COD) to be assigned. An investigation of SUDI results in two cohorts: (i) those with an identified COD and (ii) those where the death remains unexplained. Unexplained deaths that appear to have a natural cause may be classified as Sudden Infant Death Syndrome (SIDS) as a diagnosis of exclusion. In the UK, approximately 40% of SUDI remain unexplained after standard autopsy investigation, including microbiological sampling (Office for National Statistics, 2017). Families who receive this diagnosis experience greater guilt and self-blame, resulting in a more difficult and prolonged grieving process (Carlson, 1993; Garstang et al., 2016).

Bacterial infection contributes to a significant identified cause of SUDI (Weber et al., 2010; Pryce et al., 2011; Goldwater, 2017). These infections often have a sudden onset and rapid deterioration of the infant’s health. To diagnose an infectious cause of SUDI, pathologists require either clear histological evidence of infection/inflammation withe the diagnosis confirmed with the identification of a causative pathogen (Pryce et al., 2011). However, in the circumstances of a sudden unexpected death, post-mortem (PM) findings may be difficult to interpret due to changes that may occur after death (Rambaud et al., 1999; Pryce et al., 2011; Fernández-Rodríguez et al., 2019).

To investigate infection as a COD, tissues are routinely sampled at autopsy for microbiology investigation. However, PM circumstances make interpretation of results notoriously difficult since positive PM cultures could represent true antemortem infection caused by the identified organism, contamination from sample collection and handling, or artifact resulting from the PM decomposition process. Although contamination during sample collection and handling can be reduced using an aseptic technique, contamination due to natural decomposition is more difficult to control. PM translocation is the spread of viable bacteria from sites of high colonization to extra-intestinal tissue following death and occurs due to body decomposition (Mesli et al., 2017). In animal models in the forensic context at ambient temperatures, this process commences as rapidly as 5 minutes after death (Heimesaat et al., 2012; Burcham et al., 2016). However, keeping the carcass refrigerated between death and PM examination is thought to limit the extent of PM translocation (Morris et al., 2007; Lawrence et al., 2019). Recent data suggest that PM translocation can be characterized in animal models kept under clinical conditions using 16S rRNA gene sequencing (Gates et al., 2021). The ability of this technique to characterize diverse microbial communities has the potential to assist in the interpretation of PM microbiological findings.

At present, microbial culture remains the gold standard method to investigate infection as a cause of SUDI. This method requires microbes to be cultured on selective media under laboratory conditions and is therefore biased toward readily culturable microbes. False-negative results may also be obtained in cases where microbes cannot be cultured due to complex condition requirements not replicated in the laboratory or being moribund at the time of sampling due to antimicrobial treatment, as is often the case in SUDI. Thus, culture techniques are associated with many limitations that may impede the recovery of pathogens at PM investigation.

The limitations of bacterial culture in other clinical settings have been countered with the use of molecular methods such as species-specific polymerase chain reaction (PCR) (Shang et al., 2005; Rampini et al., 2011; Liu et al., 2014; Aggarwal et al., 2020). These methods require an initial indication of the infection-causing pathogen. In cases where the infection is perhaps caused by rare bacteria, as may be the case in SUDI, the infection-causing pathogen may remain undetected. Hence, there is a demand for a more unbiased approach that can target all bacteria within a sample.

16S rRNA gene sequencing targets the 16S rRNA gene present in all bacterial species. The gene is highly conserved and has a unique structure that allows the DNA sequence to act as a fingerprint for different bacterial species. Sequencing of these unique regions allows the identification of bacteria using reference databases such as Greengenes, RDP, and SILVA (McDonald et al., 2012; Cole et al., 2014; Yilmaz et al., 2014). By performing targeted high-throughput DNA sequencing of the 16S rRNA gene in PM samples, we can obtain detailed information regarding bacterial DNA present, as well as relative abundances of identified bacteria assisting in determining the relevance of findings. Applying this technique to PM tissues obtained from cases could be implemented in the clinic to enhance PM diagnosis of infection and therefore reduce the number of cases that remain unexplained.

This study aimed to investigate whether 16S rRNA gene sequencing could be used to detect bacterial infections in PM tissues collected as a part of routine PM investigation of sudden unexpected death in infancy and childhood (SUDIC). We hypothesized that the 16S rRNA gene sequencing technique would be superior to current culture techniques for the detection of bacteria in PM tissues, thus improving PM diagnosis of bacterial infection.

## Methods

### Case selection

Cases of SUDIC that had undergone extensive autopsy between 2011 and 2019 were identified from the diagnostic archive at Great Ormond Street Hospital (GOSH). In all cases, consent for research had been provided by the parents, and the study was approved by the Research Ethics Committee. All cases selected for this study underwent a comprehensive autopsy at the centre according to a standard protocol investigated on behalf of Her Majesty’s Coroner (HMC). Cases were selected based on their final diagnosis and grouped into one of three groups based on the final COD: (Weber et al., 2012) explained non-infectious, (Kennedy, 2016) infectious, and (Office for National Statistics, 2017) unexplained. Archival frozen material was collected for each case where available and included the heart, kidney, liver, spleen, and muscle samples. Frozen lung tissue was not available. All cases were deidentified and allocated a study number only. Available clinical information was provided at the autopsy.

### DNA extraction and sequencing

An approximately 1 mm^2^ section of each frozen tissue was cut using a sterile scalpel in a sterile Petri dish kept on dry ice. The tissue was then digested by adding 180 μL ATL buffer (Qiagen) and 20 μL Proteinase K (Qiagen) and incubated at 56°C overnight. DNA was then extracted using the QIAamp DNA Mini Kit (Qiagen) following the manufacturer’s protocol with the addition of an initial lysing step using Ribolysing Matrix B (MP Biomedicals) and bead-beating for 1 min at 50 oscillations/s. DNA templates were amplified for sequencing using the Taq PCR Core Kit (Qiagen) together with primers targeting the V3–V4 region of the 16S rRNA gene. These primers had adapters and indexes attached as previously described (Rosser et al., 2020). A ZymoBIOMICS Microbial Community Standard (Zymo Research) of known bacterial composition was used to assess bias and sequencing error rates. Following amplification, PCR products were subject to size selection using Agencourt AMPure XP beads (Beckman Coulter) by adding 0.7 μL beads per 1 μL of PCR product. The post-PCR clean-up DNA concentration was measured using the Qubit dsDNA High Sensitivity kit (Invitrogen). A DNA concentration of <0.2 ng/μL was considered negative. Amplification of each DNA extract was performed thrice before being considered negative. Positive samples were normalized to the desired library concentration of 0.3 ng/μL using nuclease-free water, pooled, and sequenced on the MiSeq platform using a 500-v2 cartridge (Illumina).

### Data processing and statistical analysis

Sequencing data were demultiplexed on the MiSeq platform. Paired-end reads were merged using FLASH (version 1.2.11) (Magoč and Salzberg, 2011) and quality filtered using VSEARCH (Rognes et al., 2016). Quality filtering included trimming, alignment, removal of sequences with greater than four expected errors, and identification and removal of suspected chimeras. Taxonomic classification of generated operational taxonomic units (OTUs) was carried out on the Mothur platform (version 1.44.0) (Schloss et al., 2009) using the RDP reference database (version 16, released in 2016) (Cole et al., 2014) with a similarity cut-off of 97%. Output files were analyzed using R Studio (version 1.2.5001). Samples with quality filtered sequencing reads within two standard deviations from the mean number of reads within the negative extraction control samples were removed. The mock community was assessed for any unexpected, spurious OTUs, and abundances were used as a cut-off for sequencing artifacts. Statistical analysis was performed using GraphPad Prism (version 9.3.1). Fisher’s exact test was used to compare positive samples between the COD groups, for which a *p*-value < 0.05 was considered significant. The Shannon entropy was calculated for each PM tissue to gain a numerical representation of the microbial diversity and structure of each bacterial community. Figures were generated using GraphPad Prism (version 9.3.1).

### Species-specific qPCR

In PM tissues where a dominant bacterium was identified using 16S rRNA gene sequencing, we used qPCR reactions for the most observed bacterial pathogens at GOSH causing infection in this patient population, which have been previously validated in the clinical lab. The qPCR reactions were performed as follows: 12.5 μL of 2x QuantiTect Probe PCR Mastermix (Qiagen), 0.2 μM of forward primer, 0.2 μM of reverse primer, 0.2 μM of probe, and 2 μL of DNA extract, made up to 25 μL with nuclease-free H2O. The reactions were performed on a BioRad CFX96 real-time system with the following cycling conditions: an initial 15 min at 95°C, followed by 45 cycles of 94°C for 15 s, and 60°C for 60 s. Each reaction included a positive control and a blank negative control. The primer sequences and positive controls for each species are given in Supplementary Data 1.

## Results

In total, 42 cases of SUDIC were used in this study. Frozen PM tissues included the heart, kidney, liver, muscle, and spleen samples (*n* = 187). Each case of SUDIC was categorized into one of three groups depending on the final COD as stated on the final PM report; (Weber et al., 2012) explained non-infectious (*n* = 14), (Kennedy, 2016) infectious (*n* = 8), and (Office for National Statistics, 2017) unexplained (*n* = 20). Frozen PM tissues were obtained for each case, and the time between death and autopsy, or the post-mortem interval (PMI), is shown in Supplementary Data 2. The COD in the explained non-infectious cohort included, for example, severe head injury, allergic inflammation, congenital heart disease, and metastatic tumor. The infectious death cohort included five cases of clinical and microbiological septicemia and three cases of respiratory infection.

Of the 187 frozen PM tissue samples, 66 (35%) were positive for the bacterial 16S rRNA gene using PCR. The number of PCR-positive samples in each COD group is shown in Table 1. The infectious group had the greatest proportion of tissues considered positive (68%) compared to the explained non-infectious (20%) and unexplained (33%) groups (*p* < 0.0001 and *p* = 0.0006, respectively). There was no correlation between the positive tissue samples and the PMI.

**Table 1:** Number of samples positive for the 16S rRNA gene in each cause of the death group.

Group	Total samples	Positive	Negative	Group comparison
(1) Explained, non-infectious	59	12 (20%)	47 (80%)	(1) vs. (2) *P* < 0.0001
(2) Infectious	34	23 (68%)	11 (32%)	(2) vs. (3) *P* = 0.0006
(3) Unexplained	94	31 (33%)	63 (67%)	(1) vs. (3) *P* = 0.0997

Group comparison statistics were calculated using Fisher’s exact test. A *P*-value < 0.05 was considered significant.

**Figure 1:**
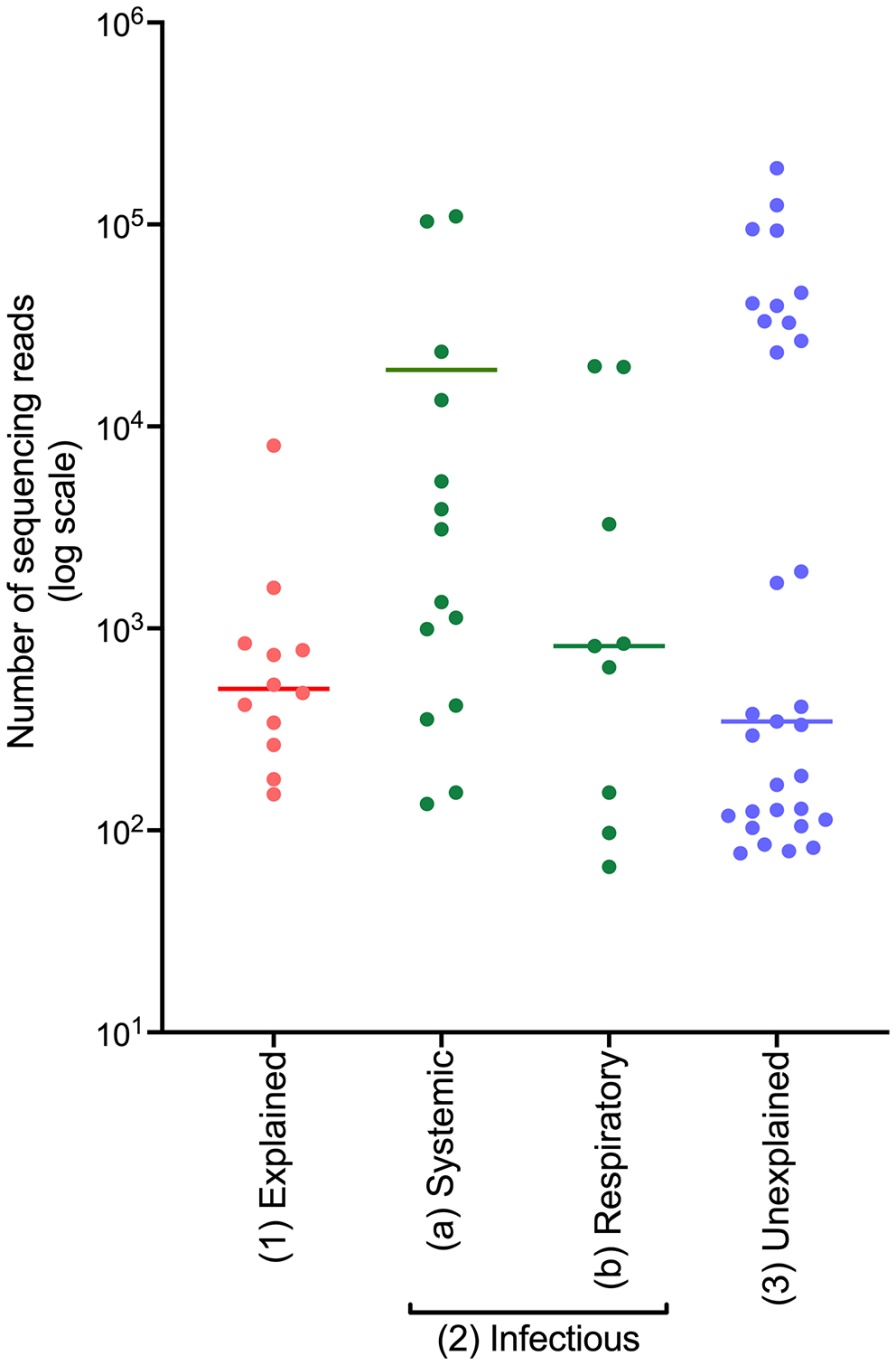
The number of sequencing reads generated from frozen PM tissues from SUDIC that were positive for the 16S rRNA gene. The number of sequencing reads is shown on a logarithmic scale. The line represents the median number of reads.

On initial observation of the generated sequencing data, a difference in the number of sequencing reads generated within each COD group was observed (displayed on a logarithmic scale in Figure 1). The average read numbers for the explained non-infectious, infectious, and unexplained groups were 1,177, 12,026, and 16,695, respectively. Cases from the infectious cohort were split into two groups: (i) systemic infection and (ii) respiratory infection, since frozen lung tissue was not available and respiratory infections are often localised meaning the bacteria may not be detected in other tissues. PM tissues from the cases with systemic infection had a greater average number of sequencing reads (19,076) compared to those from the cases of respiratory infection (4,552).

### Systemic infection

To investigate whether this technique could successfully identify the bacterial pathogen in SUDIC caused by bacterial infection, we examined results obtained from the systemic infectious group (Table 2; relative abundance plots presented in Supplementary Data 3A). In 2/5 (40%) cases, the bacterial pathogen could not be identified at routine PM using bacterial culture. However, a likely pathogen was identified in at least one PM tissue from all cases using 16S rRNA gene sequencing. In all cases where the bacterial pathogen had been identified and included in the PM report, this was the dominant finding in the corresponding case by 16S rRNA gene sequencing. In one case, the causative organism could not be cultured from the tissues collected at autopsy, but a blood sample collected on arrival at the emergency department revealed *Neisseria meningitidis*. In another case, the bacterial pathogen was not identified but the autopsy report stated that the histological changes were suggestive of sepsis caused by *Streptococcus pneumoniae*, Group A Streptococcus, or *Haemophilus influenzae*. In this case, 16S rRNA gene sequencing identified *Streptococcus* in the PM tissue.

**Table 2:** Findings from cases of SUDIC with systemic infection from routine investigation and following 16S rRNA gene sequencing and species-specific qPCR.

Case	Relevant clinical notes	Organisms identified during PM microbiological investigation (site)	Organisms identified in tissues passing defined threshold (site)	Bacterial species identified by qPCR
i1	Fever Cough Runny nose Vomiting	**Group A Streptococcus** (blood, CSF, lung, spleen)	***Streptococcus*** (spleen)	**Group A Streptococcus**
i2^1^	Fever GP visit Antibiotic administration	Negative (blood, lung, spleen, CSF) *Neisseria meningitidis* in ante-mortem blood by PCR (not part of routine testing)	***Neisseria*** (heart, kidney, liver) Enterococcus (muscle)	*Neisseria meningitidis*
i3	Fever Cough Runny nose Diarrhea GP visit	*Neisseria meningitidis* (blood, CSF, lung) URT commensals (spleen)	***Neisseria*** (heart, liver, spleen) *Enterococcus* (kidney)	*Neisseria meningitidis*
i4^1^	Fever Cough Vomiting Antibiotic administration Histology consistent with septicaemia caused by *Streptococcus pneumoniae*, GAS^2^, and *Haemophilus influenzae*	Negative (blood, lung, CSF)	***Streptococcus*** (heart) Negative (kidney, liver, muscle, spleen)	*Streptococcus pneumoniae*
i5	Fever Vomiting Throat infection GP visit Antibiotic administration	*Klebsiella pneumoniae* (blood, tonsils, lung, spleen) Negative (CSF)	**Enterobacteriaceae** (kidney, liver, spleen, muscle) Negative (heart)	Not performed

^1^Cases where a bacterial pathogen was not identified at routine investigation using bacterial culture, but where a likely causative organism was identified in the present study.

^2^Group A Streptococcus. Bacterial species in bold were determined to be the infection-causing bacteria at routine PM investigation.

The DNA sequence of the V3–V4 region is unable to classify bacteria to the species level with >97% confidence. Therefore, bacteria identified in this study could only be classified to genus level. For a pathologist to reliably interpret results, species classification is preferable. Therefore, in samples where a dominant bacterium was identified, species-specific qPCRs were performed in a hybrid approach (Table 2). In all cases where a species-specific PCR was performed and a species had been identified by PM microbiology, the same bacterium was identified by PCR.

### Respiratory infection

Relative abundance plots displaying 16S rRNA gene sequencing findings from the three cases of SUDIC caused by respiratory infection are shown in Supplementary Data 3B. Two were due to bacterial infection and one was of viral origin as stated in the final PM reports. In the two cases of bacterial infection, the causative organism remained unidentified at routine PM investigation and was identified based on histological findings.

### Explained non-infectious death

The explained non-infectious cohort consisted of 14 cases. Relative abundance plots are presented in Supplementary Data 4. Generally, PM tissues from this group generated a lower number of sequencing reads and frozen PM tissue samples displayed mixed bacterial communities, rather than a single dominating OTU. The bacteria identified within these tissues included *Streptococcus*, *Faecalibacterium*, Ruminococcaceae, Lachnospiraceae, *Roseburia*, and *Veillonella*.

**Figure 2:**
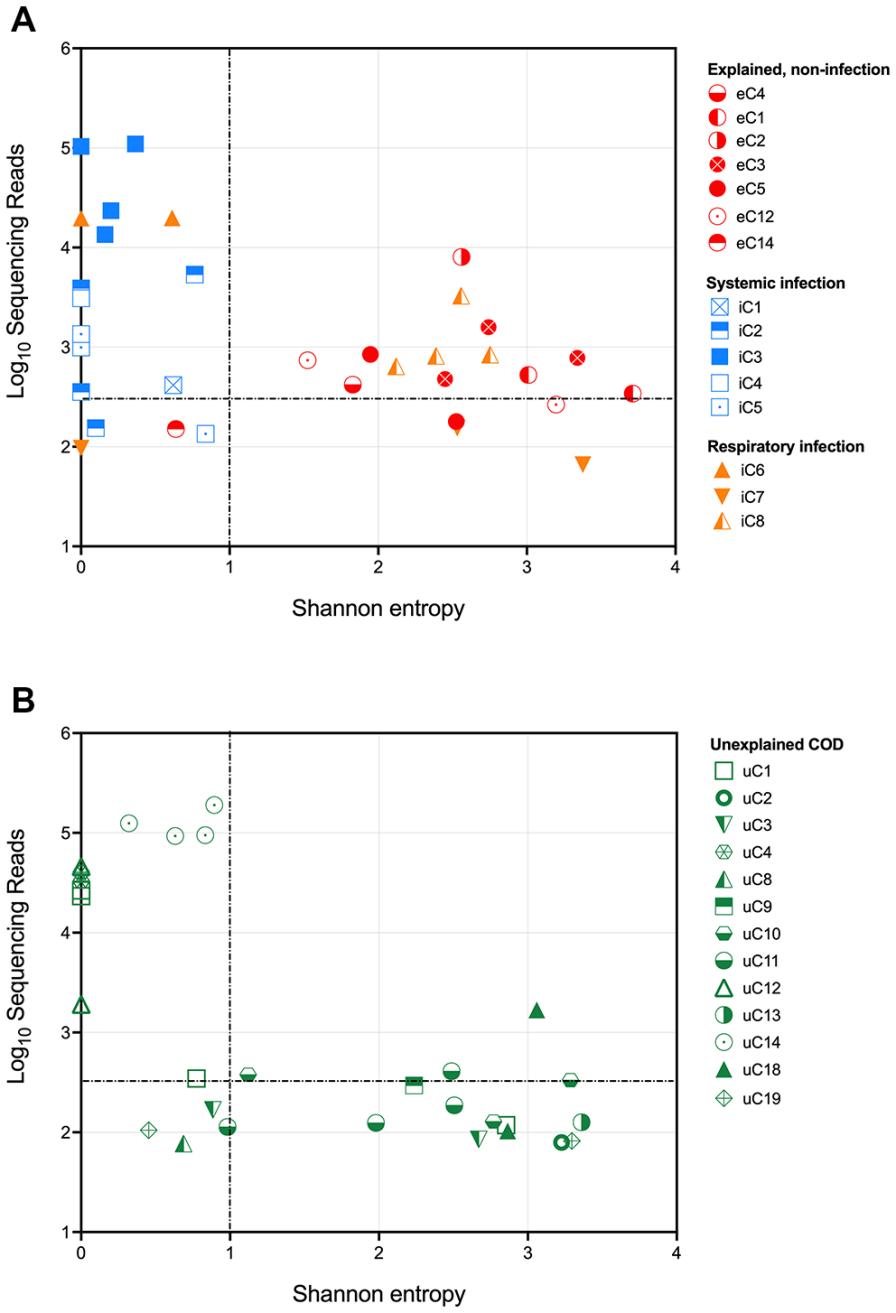
Shannon entropy and log_10_[sequencing reads] for each PM tissue from **(A)** SUDIC with an identified cause of death and **(B)** SUDIC that remained unexplained following a routine investigation. Dotted lines show where criteria were defined. The colors show the final COD and the shapes represent the case number. PM tissues that are in the upper left-hand quadrant are likely due to a bacterial infection.

The results from the infectious and explained non-infectious groups demonstrate that 16S rRNA gene sequencing could successfully distinguish between the cohorts by assessing both the total number of sequencing reads and the community structure. Shannon entropy was calculated for each sample to numerically represent community diversity (Figure 2A). Cases with a known systemic infection clustered on the upper left-hand side of the plot, demonstrating low Shannon entropy and a high number of sequencing reads. PM samples from cases with an explained, non-infectious COD cohort clustered on the lower right-hand side of the plot, demonstrating a lower number of sequencing reads and greater diversity. PM samples from cases with a known respiratory infection either clustered with the PM tissues from infectious deaths or those with an explained non-infectious COD. This is likely due to some respiratory infections remaining localized and the unavailability of frozen lung tissue. Following visualization of the results, we established thresholds to identify cases with a likely bacterial infection (dotted lines in Figure 2). These were set based on clear distinguishers between the two groups with a known COD. For tissue to be considered infected, we defined a threshold of >350 sequencing reads and a Shannon entropy measure of <1.

Applying these thresholds to our sequencing data, we found that 14/24 (58%) PM tissue samples collected from 5 cases of systemic infection and 3 cases of respiratory infection were likely to have been infected at the time of death. At least one PM tissue from each of the 5 cases of respiratory infection was considered positive for bacterial infection. Of the 3 cases of respiratory infection, only one case contained positive PM tissues.

Our method and defined thresholds allowed us to identify all cases of systemic infection. We did not identify any cases with an explained, non-infectious COD and therefore had no false-positive results. We were unable to detect cases of respiratory infection in for this study due to the unavailability of PM lung tissue.

### Unexplained cohort

To investigate whether any currently unexplained SUDIC may have been due to a possible undetected systemic infection, these samples were subjected to the above thresholds (Figure 2B). A total of 33 tissues were positive, of which 12 (36%) passed the defined threshold. These 12 samples were obtained from 4/20 cases of SUDIC suggesting that up to 20% of currently unexplained SUDIC may be due to systemic infection previously undetected using standard methods (Table 3 and Supplementary Data 5). Clinical history was available for three of the four cases in which positive tissues were identified. For all three infants, in cases where clinical history was available, fever and irritability were noted as consistent with possible infection. The infection-causing bacterium identified in these cases included *Clostridium sordellii*, *Enterococcus faecalis*, *Enterococcus faecium*, and *Enterobacteriaceae*.

**Table 3:** Results from routine investigation and 16S rRNA gene sequencing and species-specific qPCR from unexplained SUDIC.

Case	Relevant clinical notes	Organisms identified during PM microbiological investigation (site)	Organisms identified in tissues passing defined threshold (site)	Bacterial species identified by qPCR
1	Hospitalization due to bronchiolitis Fever Irritability	URT commensals (lung) Negative (spleen)	***Enterococcus*** (heart spleen)	*Enterococcus faecium*
2	No appetite Fever Irritability Change in breathing	Negative (spleen, lung)	***Enterococcus*** (heart, kidney, liver, muscle) Enterobacteriaceae sp. (heart, kidney, liver, muscle)	*Enterococcus faecalis*
3	No clinical history available	*Clostridium* species (blood, spleen) *Staphylococcus aureus* (lung)	***Escherichia****/Shigella* (liver, muscle)	*Escherichia coli*
4	Fever Irritability	*Clostridium sordellii* (lung, swabs from auditory canals) *Actinomyces lingnae* (blood)	***Clostridium*** (heart, kidney, liver, muscle)	Not performed

Bacterial species in bold were determined to be the infection-causing bacteria at routine PM investigation. *URT = upper respiratory tract.

## Discussion

This study has demonstrated for the first time that 16S rRNA gene sequencing can be used in PM tissue to identify bacterial infections that may have caused SUDIC. The technique provides a more objective approach than the current culture methods with clearer results less affected by interpretation bias. The method was unaffected by potential contamination of both internal and external origin and could therefore enhance PM investigation of bacterial infection in cases of SUDIC. Specifically, 16S rRNA gene sequencing of PM tissues allows for highly reliable distinguishing between infectious and non-infectious deaths and suggests that approximately 20% of currently unexplained SUDIC deaths could represent undetected infectious causes.

Over the last decade, 16S rRNA gene sequencing has been investigated under many clinical circumstances and has repeatedly been shown to identify more organisms than culture alone (Rhoads et al., 2012; Xia et al., 2015; Decuypere et al., 2016; Hashavya et al., 2018). We successfully applied this technique to PM tissues from cases of SUDIC as an initial screening approach to identify the type of bacterial genera present, which was then followed by a more targeted molecular approach to confirm the infection-causing species.

We were able to successfully distinguish between SUDIC with systemic infection and explained non-infectious COD. Establishing clear and simple criteria to differentiate between PM tissues obtained from systemic infection and the explained non-infectious COD group allowed all systemic infections to be successfully identified. In addition to confirming the bacterial pathogen in cases where the organism was identified at PM investigation, our investigation was also able to identify the causative organism in cases in which a definite bacterial pathogen could not be identified using routine PM microbiology. These cases had both received antibiotics prior to death, which is likely responsible for the negative PM culture, whilst 16S rRNA gene sequencing identified the bacteria. We acknowledge that in cases of systemic infection, not all PM tissues passed the threshold for a true bacterial infection, but rather at least one PM tissue from each case. We hypothesize that this is due to not all tissues being infected at the time of death or the tissue being sampled from a non-infected region.

In cases of respiratory infection, we were unable to identify a causative pathogen likely due to the unavailability of frozen lung tissue. With lung tissue available, this technique also has the potential to identify cases of localized but severe bacterial respiratory infection, which we believe has likely given the prevalence of these infections in this population. We, therefore recommend that lung tissue be routinely collected and stored as frozen specimens, where permission is granted, along with other tissues.

Contamination has typically resulted in difficulty when interpreting PM microbiology results produced by culture given the number of scenarios from which a positive culture can arise together with the generation of only qualitative results (Pryce et al., 2011; Riedel, 2014; Fernández-Rodríguez et al., 2019). However, contamination and true bacterial infection were not difficult to distinguish in this study. Analysis of the 16S rRNA gene sequencing results revealed clear differentiation between cases of systemic infection and those from other known causes. Rather than a single dominating bacterium, PM tissues that were not from infected cases of SUDIC, showed mixed bacterial communities with a relatively low number of sequencing reads. Therefore, bacteria present in tissues as a result of the PM setting did not interfere with the interpretation of bacteria present as a result of infection.

There is also a prevailing belief that PM bacterial translocation and overgrowth are responsible for the identification of common GI tract bacteria in the PM setting, further contributing to the difficulty in interpreting PM microbiology results. Although this is problematic when culturing bacteria given the ease of culturing common intestinal bacteria, together with the absence of abundance information, using a universal 16S rRNA gene sequencing approach PM bacterial translocation and overgrowth seems to be both minimal and predictable, as we have previously shown in two animal models (Gates et al., 2021). In both explained and infectious cases, the lack of or low-level identification of bacteria suggests that although this process may occur, it is not extensive and appears to involve a mixed intestinal flora, rather than a single bacterium. Therefore, the use of this method would allow better differentiation between findings of true infection versus findings as a consequence of the PM process.

Investigation of currently unexplained deaths revealed four cases that we believe are likely due to bacterial infection. The bacteria identified in the PM tissues are all common pathogens that are known to cause disease, particularly in populations with weakened or immature immune responses. The clinical notes for each of these cases revealed that they had been experiencing symptoms such as cough, fever, and irritability, all suggestive of infection. Providing 16S rRNA gene sequencing results in addition to all other PM investigative results may have assisted in the interpretation of the SUDIC autopsy report and ultimately lead to a diagnosis, thus reducing the number of unexplained cases.

To conclude, this study has provided supporting evidence for the use of 16S rRNA gene sequencing in the PM investigation of SUDIC. There were two main findings of this study that are critical to support the further exploration and potential integration of this technique into a clinical setting; (Weber et al., 2012) the successful identification of all cases of SUDIC with systemic infection and (Kennedy, 2016) the lack of false positives within the explained non-infectious group. Establishing clear and simple criteria to differentiate between bacteria present due to contamination and those that are representative of a true bacterial infection could be implemented into routine PM microbiology and would enhance the PM investigation of the infection.

## Data availability statement

The original contributions presented in the study are publicly available. This data can be found here: NCBI SRA repository (https://www.ncbi.nlm.nih.gov/sra), accession number PRJNA977699.

## Ethics statement

The studies involving human participants were reviewed and approved by the NRES Committee London - Bloomsbury. Written informed consent to participate in this study was provided by the participants’ legal guardian/next of kin.

## Author contributions

NS and NK proposed the conceptual idea for this study and the clinical need for this investigation. LG, NS, NK, and DA devised a study plan. LG identified cases suitable for investigation, confirmed consent for research, performed the remaining experimental work, and developed bioinformatic pipelines for data analysis and visualization. LG, TM, OO, and K-AK collected samples from clinical archives. LG wrote the manuscript with consultation from NS, NK, DA, TM, OO, and K-AK. All authors contributed to the article and approved the submitted version.

## Funding

This study was supported by the Lullaby Trust (Grant no. 175614). DA was funded by the Reuben Foundation.

## Conflict of interest

The authors declare that the research was conducted in the absence of any commercial or financial relationships that could be construed as a potential conflict of interest.

## Publisher’s note

All claims expressed in this article are solely those of the authors and do not necessarily represent those of their affiliated organizations, or those of the publisher, the editors and the reviewers. Any product that may be evaluated in this article, or claim that may be made by its manufacturer, is not guaranteed or endorsed by the publisher.
