# Supplementary material for: Identification of bacterial pathogens in sudden unexpected death in infancy and childhood using 16S rRNA gene sequencing
Source: Front Microbiol. 2023 Jun 15;14:1171670. doi: 10.3389/fmicb.2023.1171670 (PMC10309030; doi:10.3389/fmicb.2023.1171670)
Supplement: Supplementary file 1 [file Data_Sheet_1.DOCX]

**SUPPLEMENTARY DATA 1**

Positive control organisms were obtained as follows. The following reagents were obtained through BEI Resources, NIAID, NIH: *Enterococcus faecalis*, Strain B3286, NR-31886; Genomic DNA from *Neisseria meningitidis*, Strain 2007056, NR-48804; *Enterococcus faecium*, Strain UAA945, NR-32094; *Streptococcus pyogenes*, Strain ABC020063118, NR-48702; *Streptococcus agalactiae*, Strain SGBS001, NR-44125; *Streptococcus pneumoniae*, Strain OREP4, NR-51851.

Primer sequences for each species-specific qPCR are given below.

| Target species | Primer | Sequence (5' to 3') |
| --- | --- | --- |
| *Enterococcus faecalis* | Forward | GAC AGG AAA GAA ACT AGG AGG AC |
|  | Reverse | AAA CAG ACA CAT CGT GCT |
|  | Probe | [Cy5]CAC TTC TGC CGC CAT ACA ACA A[BHQ3] |
| *Enterococcus faecium* | Forward | CGT AGC ATT CTA TGA TTA TGA AGC C |
|  | Reverse | CAT CGT GTA AGC TAA CTT CG |
|  | Probe | [6FAM]CAG ATT CCA GCC GAA GTG CC[TAM] |
| Group B Streptococcus | Forward | ATC CTG AGA CAA CAC TGA CA |
|  | Reverse | TTG CTG GTG TTT CTA TTT TCA |
|  | Probe | [JOE]ATC AGA AGA GTC ATA CTG CYA CTT C[BHQ1] |
| *Neisseria meningitidis* | Forward | GCT GCG GTA GGT GGT TCA A |
|  | Reverse | TTG TCG CGG ATT TGC AAC TA |
|  | Probe | [JOE]CAT TGC CAC GTG TCA GCT GCA CAT[BHQ1] |
| *Streptococcus pneumoniae* | Forward | ACG CAA TCT AGC AGA TGA AGC |
|  | Reverse | TGT TTG GTT GGT TAT TCG TGC |
|  | Probe | [FAM]TTT GCC GAA AAC GCT TGA TAC AGG G[BHQ-1] |
