# Supplementary material for: Identification of bacterial pathogens in sudden unexpected death in infancy and childhood using 16S rRNA gene sequencing
Source: Front Microbiol. 2023 Jun 15;14:1171670. doi: 10.3389/fmicb.2023.1171670 (PMC10309030; doi:10.3389/fmicb.2023.1171670)
Supplement: Supplementary file 2 [file Data_Sheet_2.DOCX]

| Case | Post-mortem  interval (days) | Frozen tissues obtained |
| --- | --- | --- |
| eC1 | 3 | H, K, Li, M, S |
| eC2 | 8 | H, K, Li, M, S |
| eC3 | 4 | H, K, Li, M, S |
| eC4 | 1 | H, K, Li, M, S |
| eC5 | 2 | H, K, Li, M, S |
| eC6 | 1 | H, K |
| eC7 | 6 | H, K, Li, M, S |
| eC8 | 1 | H, K |
| eC9 | 1 | H, K, Li, M, S |
| eC10 | 9 | H, K, Li, S |
| eC11 | 4 | H, K, Li, M, S |
| eC12 | 4 | H, K, Li, M, S |
| eC13 | 1 | S |
| eC14 | 3 | H, K, Li, M, S |
| iC1 | 5 | S |
| iC2 | 5 | H, K, Li, M, S |
| iC3 | 3 | H, K, Li, M, S |
| iC4 | 4 | H, K, Li, M, S |
| iC5 | 3 | H, K, Li, M, S |
| iC6 | 4 | H, K, Li, M |
| iC7 | 6 | H, K, Li, M, S |
| iC8 | 7 | H, K, Li, M |
| uC1 | 3 | H, K, Li, M, S |
| uC2 | 5 | K, Li, M, S |
| uC3 | 7 | H, K, Li, S |
| uC4 | 6 | H, K, Li, M |
| uC5 | 5 | H, K, Li, M, S |
| uC6 | 4 | H, K, Li, M, S |
| uC7 | 2 | H, K, Li, M, S |
| uC8 | 11 | H, K, Li, M, |
| uC9 | 5 | H, K, Li, M, S |
| uC10 | 3 | H, K, Li, M, S |
| uC11 | 4 | H, K, Li, M, S |
| uC12 | 4 | H, K, Li, S |
| uC13 | 5 | H, K, Li, M, S |
| uC14 | 2 | H, K, Li, M |
| uC15 | 3 | H, K, Li, M, S |
| uC16 | 7 | H, K, Li, M, S |
| uC17 | 1 | H, K, Li, M, S |
| uC18 | 1 | H, K, Li, M, S |
| uC19 | 1 | H, K, Li, M, S |
| uC20 | 4 | H, K, Li, M, S |

**SUPPLEMENTARY DATA 2**

The post mortem interval and frozen PM tissues obtained from each study case. The lower-case letter refers to the PM group each case belongs to i.e. e = explained, non-infectious COD, i = infectious COD, and u = unexplained COD. The frozen tissues are as follows: H = heart, K = kidney, Li = liver, M = muscle, S = spleen.
