# Supplementary material for: Identification of bacterial pathogens in sudden unexpected death in infancy and childhood using 16S rRNA gene sequencing
Source: Front Microbiol. 2023 Jun 15;14:1171670. doi: 10.3389/fmicb.2023.1171670 (PMC10309030; doi:10.3389/fmicb.2023.1171670)

**SUPPLEMENTARY DATA 3**

Relative abundance plots for cases of SUDIC caused by (A) systemic infection (cases i1 to i5) and (B) respiratory infection (cases i6 to i8). The x-axis describes the PM tissue sampled and coloured bars represent the bacteria identified.


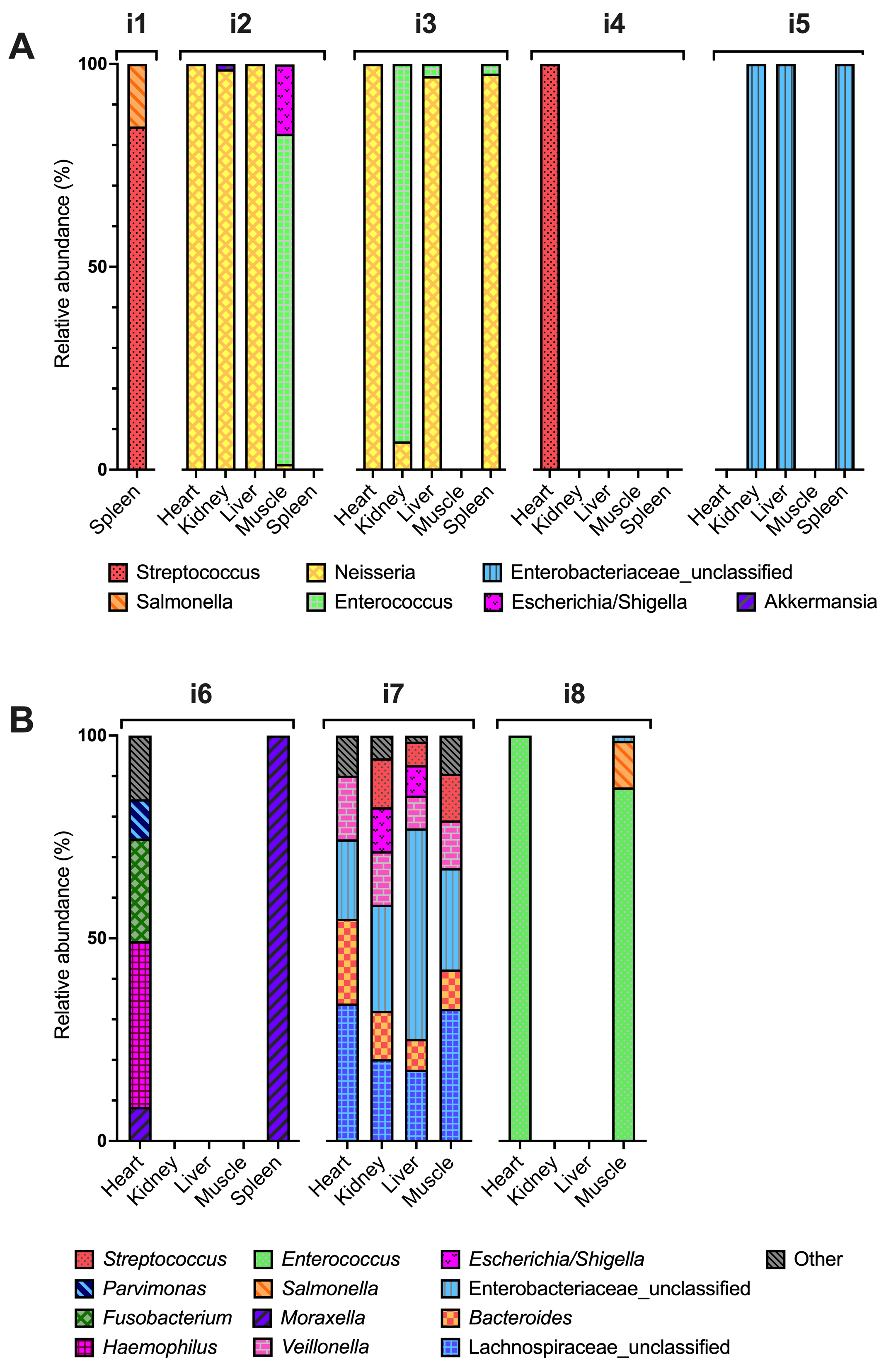

Supplement: Supplementary file 3 [file Data_Sheet_3.DOCX]
