# Supplementary material for: Identification of bacterial pathogens in sudden unexpected death in infancy and childhood using 16S rRNA gene sequencing
Source: Front Microbiol. 2023 Jun 15;14:1171670. doi: 10.3389/fmicb.2023.1171670 (PMC10309030; doi:10.3389/fmicb.2023.1171670)

**SUPPLEMENTARY DATA 4**

Relative abundance plots for cases of SUDIC caused by with an identified, non-infectious cause of death. The x-axis describes the PM tissue sampled and coloured bars represent the bacteria identified. The x-axis labels without a bar show tissues that were collected but which failed to produce 16S rRNA gene sequencing results.


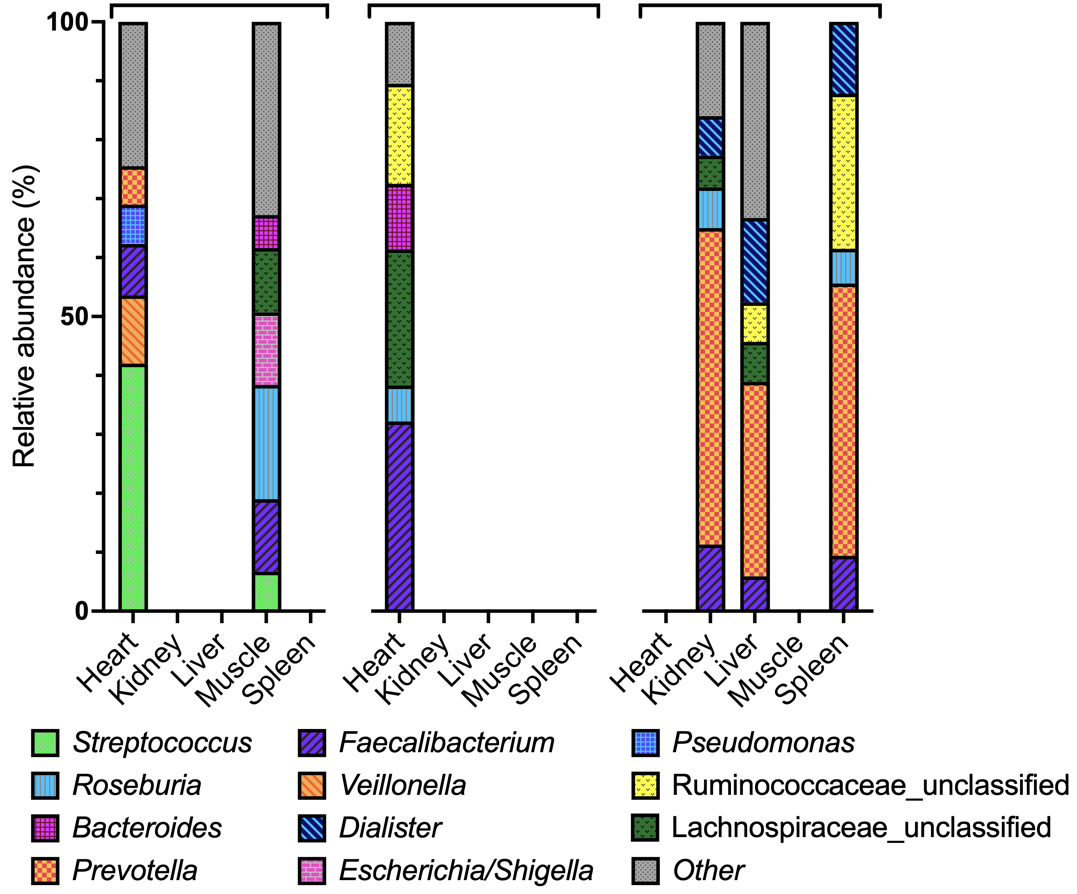

Supplement: Supplementary file 4 [file Data_Sheet_4.DOCX]
