# Supplementary material for: Identification of bacterial pathogens in sudden unexpected death in infancy and childhood using 16S rRNA gene sequencing
Source: Front Microbiol. 2023 Jun 15;14:1171670. doi: 10.3389/fmicb.2023.1171670 (PMC10309030; doi:10.3389/fmicb.2023.1171670)
Supplement: Supplementary file 5 [file Data_Sheet_5.DOCX]

**SUPPLEMENTARY DATA 5**

Relative abundance plots for cases of SUDIC with an unexplained cause (u1 to u4). The x-axis describes the PM tissue sampled and coloured bars represent the bacteria identified.
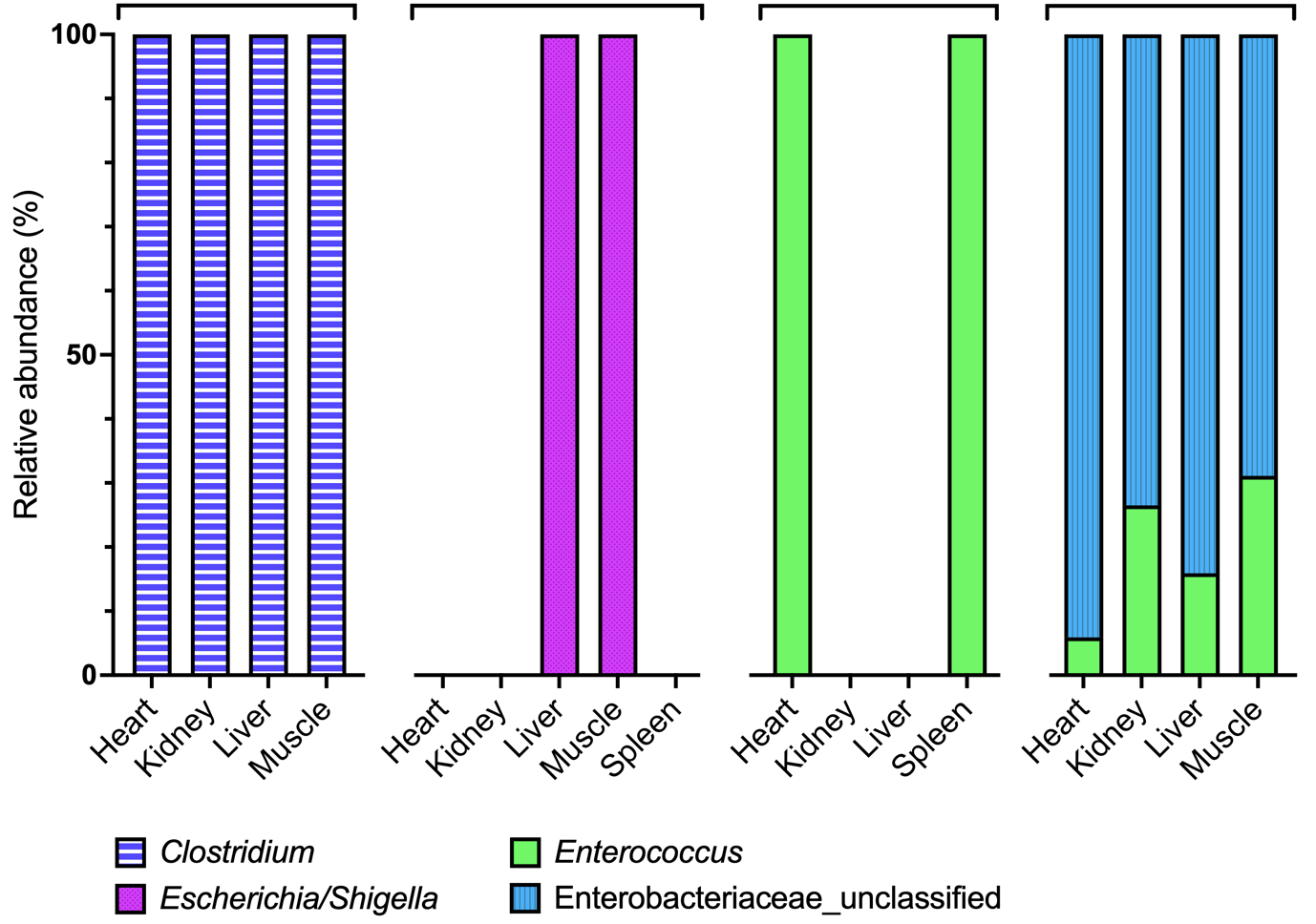


**u4**

**u3**

**u1**

**u2**
